# Crystal structure of tetra­ethyl 27,30-dioxo-7,12,20,25-tetra-*tert*-but­yl-3,16-dioxa-9,22,28,31-tetra­thia­hepta­cyclo­[21.3.1.1^1,5^.1^4,8^.1^10,14^.1^14,18^.1^17,21^]dotriaconta-4,6,8(29),10,12,17,19,21(32),23,25-deca­ene-2,2,15,15-tetra­carboxyl­ate

**DOI:** 10.1107/S205698901501748X

**Published:** 2015-09-26

**Authors:** Mehmet Akkurt, Jerry P. Jasinski, Shaaban K. Mohamed, Omran A. Omran, Mustafa R. Albayati

**Affiliations:** a Department of Physics, Faculty of Sciences, Erciyes University, 38039 Kayseri, Turkey; b Department of Chemistry, Keene State College, 229 Main Street, Keene, NH 03435-2001, USA; c Chemistry and Environmental Division, Manchester Metropolitan University, Manchester M1 5GD, England; d Chemistry Department, Faculty of Science, Minia University, 61519 El-Minia, Egypt; e Medical Laboratory Department, College of Science, Majmaah University, 11932, Saudi Arabia; f Chemistry Department, Faculty of Science, Sohag University, 82524 Sohag, Egypt; g Kirkuk University, College of Science, Department of Chemistry, Kirkuk, Iraq

**Keywords:** crystal structure, calixarenes, macrocycles, hydrogen bonding

## Abstract

The asymmetric unit of the title compound, C_54_H_64_O_12_S_4_, consists of one half of the mol­ecule, which is located on an inversion centre. The heterocyclic six-membered ring adopts a distorted envelope conformation with the spiro C atom as the flap. In the crystal, mol­ecules are linked by weak C—H⋯O hydrogen bonds with an *R* ^2^_2_(14) motif, forming a chain along the *b-*axis direction.

## Related literature   

For industrial application of calixarenes, see: Shokova & Kovalev (2003 ▸); Stoikov *et al.* (2003 ▸). For macrocyclic reactions of calixarenes, see: Agrawal & Pancholi (2007 ▸); Higuchi *et al.* (2000 ▸); Omran & Anti­pin (2014 ▸).
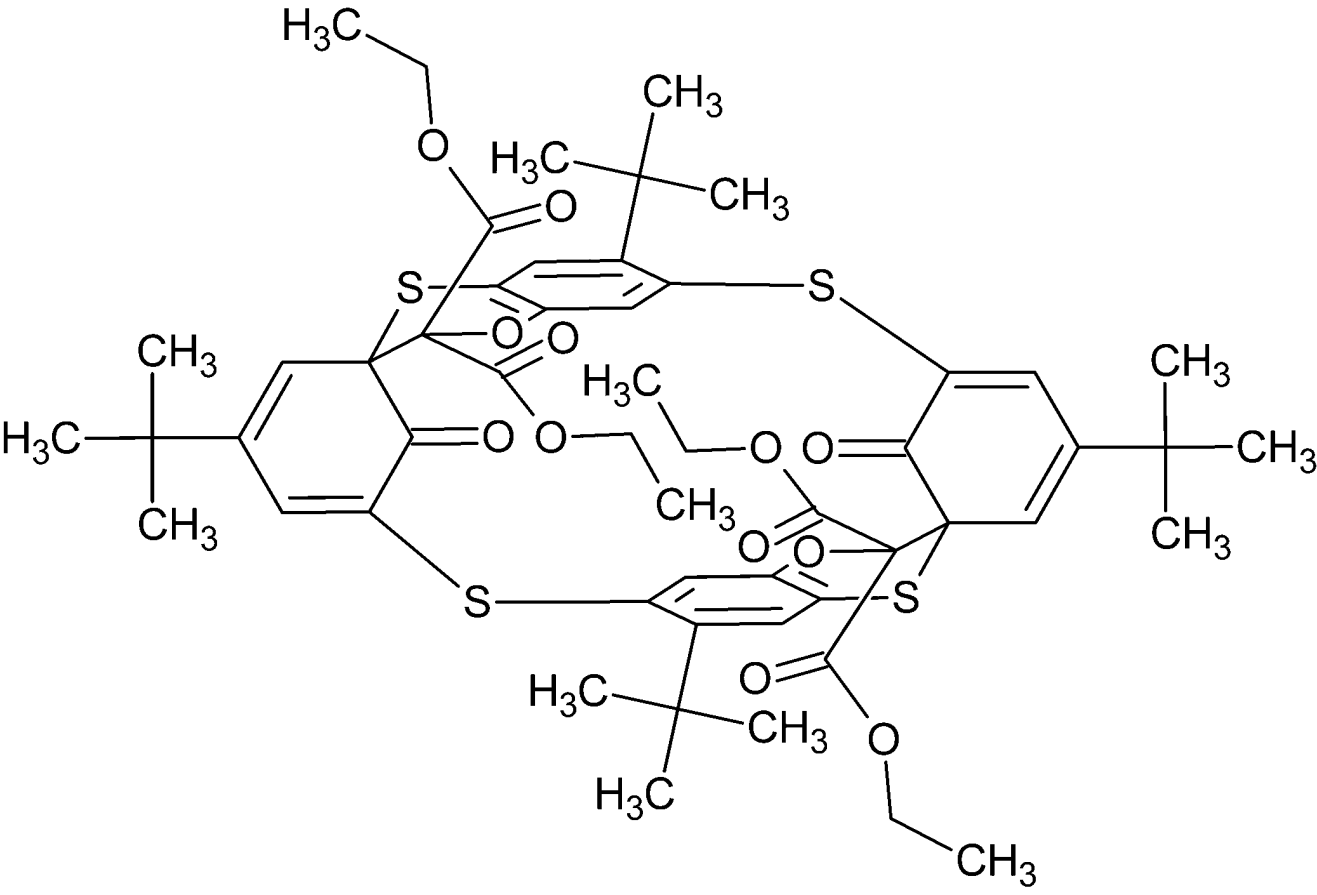


## Experimental   

### Crystal data  

C_54_H_64_O_12_S_4_
*M* *_r_* = 1033.29
Triclinic, 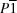

*a* = 10.9366 (6) Å
*b* = 11.7386 (5) Å
*c* = 12.6262 (8) Å
α = 100.018 (4)°
β = 113.884 (6)°
γ = 105.884 (4)°
*V* = 1348.75 (16) Å^3^
*Z* = 1
Mo *K*α radiation
μ = 0.24 mm^−1^
*T* = 293 K
0.49 × 0.32 × 0.14 mm

### Data collection  

Agilent Xcalibur, Eos, Gemini diffractometer
Absorption correction: multi-scan (*CrysAlis PRO*; Agilent, 2014 ▸) *T*_min_ = 0.715, *T*_max_ = 0.967
16365 measured reflections
8908 independent reflections
6481 reflections with *I* > 2σ(*I*)
*R*_int_ = 0.036

### Refinement  

*R*[*F* ^2^ > 2σ(*F* ^2^)] = 0.064
*wR*(*F* ^2^) = 0.169
*S* = 1.07
8908 reflections
324 parameters
H-atom parameters constrained
Δρ_max_ = 0.58 e Å^−3^
Δρ_min_ = −0.38 e Å^−3^

### 

Data collection: *CrysAlis PRO* (Agilent, 2014 ▸); cell refinement: *CrysAlis PRO*; data reduction: *CrysAlis PRO*; program(s) used to solve structure: *SHELXS2014* (Sheldrick, 2008 ▸); program(s) used to refine structure: *SHELXL2014* (Sheldrick, 2015 ▸); molecular graphics: *ORTEP-3 for Windows* (Farrugia, 2012 ▸); software used to prepare material for publication: *PLATON* (Spek, 2009 ▸).

## Supplementary Material

Crystal structure: contains datablock(s) global, I. DOI: 10.1107/S205698901501748X/is5424sup1.cif

**Fig. 1.**
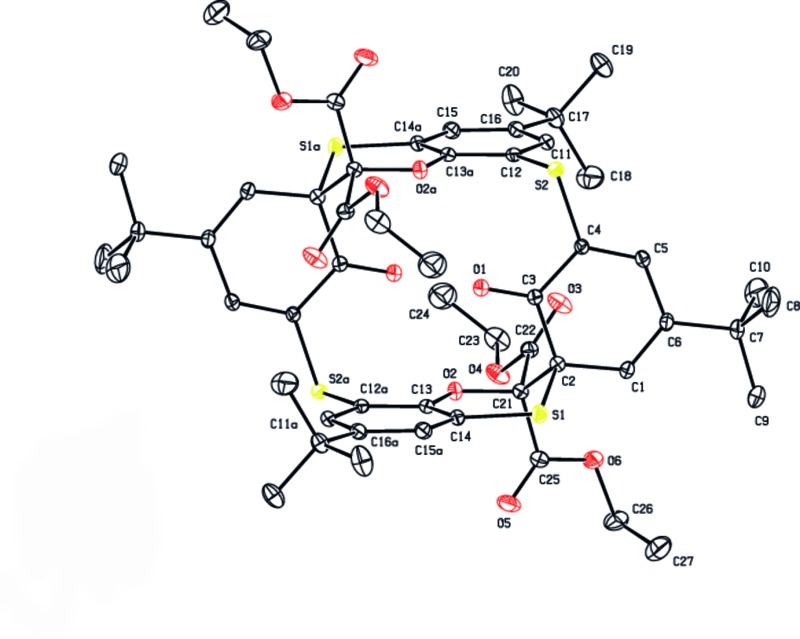
View of the title molecule with the atom-numbering scheme. Displacement ellipsoids are drawn at the 30% probability level. H atoms are omitted for clarity. [Symmetry code: (a) 1 - x, 1 - y, 1 - z.]

**Fig. 2.**
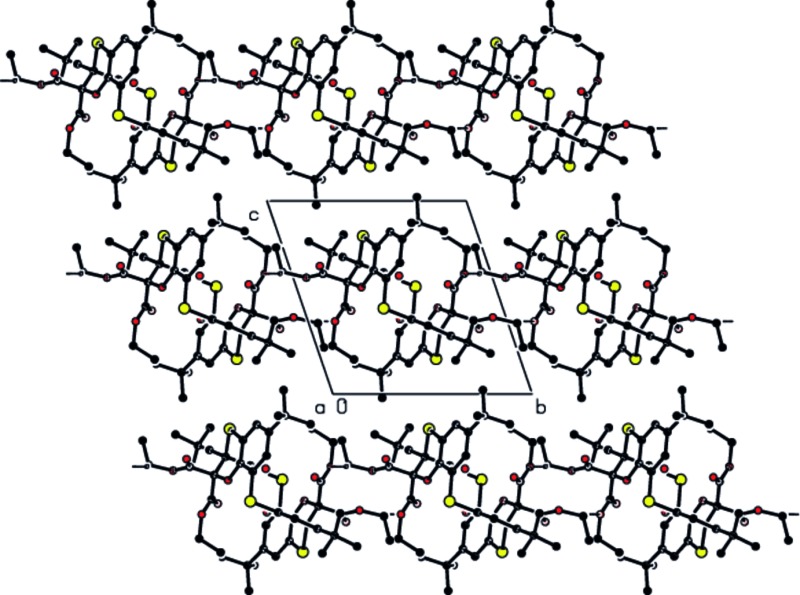
The molecular packing of the title compound viewed down the a axis. H atoms not involved in the C—H···O hydrogen bonds are omitted for clarity.

Structure factors: contains datablock(s) I. DOI: 10.1107/S205698901501748X/is5424Isup2.hkl

a x y z . DOI: 10.1107/S205698901501748X/is5424fig1.tif
View of the title mol­ecule with the atom-numbering scheme. Displacement ellipsoids are drawn at the 30% probability level. H atoms are omitted for clarity. [Symmetry code: (*a*) 1 − *x*, 1 − *y*, 1 − *z*.]

a . DOI: 10.1107/S205698901501748X/is5424fig2.tif
The mol­ecular packing of the title compound viewed down the *a* axis. H atoms not involved in the C—H⋯O hydrogen bonds are omitted for clarity.

CCDC reference: 1425819

Additional supporting information:  crystallographic information; 3D view; checkCIF report

## Figures and Tables

**Table 1:** Hydrogen-bond geometry (, )

*D*H*A*	*D*H	H*A*	*D* *A*	*D*H*A*
C26H26*A*O4^i^	0.97	2.35	3.298(5)	164

Symmetry code: (i) 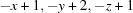.

## supplementary crystallographic information

### S1. Comment

The macrocyclic calixarenes have been considered to be not only as good substrats in industrial chemistry but also in research field of supramolecular chemistry (Agrawal & Pancholi, 2007; Higuchi *et al.*, 2000; Omran & Antipin, 2014). They have also been used as sensors, catalysis and molecular recognition or ion separtion (Shokova & Kovalev, 2003; Stoikov *et al.*, 2003). In this context and following to our on-going study we report in this study the synthesis and crystal structure of the title compound.

Figure 1 shows the title molecule which lies on a crystallographic inversion centre. The heterocyclic six-membered ring (S1/C2/C21/O2/C13/C14) adopts a distorted envelope conformation with the puckering parameters of Q_T_ = 0.547 (2) Å, θ = 50.6 (2)° and φ = 65.2 (3)°. In the crystal, weak intermolecular C—H···O hydrogen bonds (Table 1) link the molecules, forming fourteen-membered rings with *R*_2_^2^(14) ring motifs, into chains along the *b* axis (Fig. 2).

### S2. Experimental

A mixture of 1.5 g (2.08 mmol) thiacalexarene (TCA), 10 g sodium carbonate, 0.5 g of tetraethylammonium bromide (TEAB) and diethyl bromomalonate 3 ml (8. 02 mmol) in 50 ml benzene was heated at 373 K with stirring for 6 days. The reaction mixture was filtered off and the benzene layer was evaporated under vacuum to dryness. The solid residue was washed by hydrochloric diluted solution and extracted by methylene chloride (3 times). The methylene chloride portions were concentrated and methanol was added. Yellow color of two solid mixture products were precipitated and filtered off. The mixture was separated by fractional crystallization by using a mixture of methylene chloride / methanol (1:1 *v*:*v*) to afford the title compound as yellow crystals in 85% yield.

### S3. Refinement

All H atoms were placed in calculated positions with C—H = 0.93–0.97 Å and refined as riding with *U*_iso_(H) = 1.2 or 1.5*U*_eq_ (C). The (-2 5 1), (-2 5 0), (2 - 2 4), (-3 - 7 1), (-4 7 1), (1 6 0), (-5 1 2), (1 - 2 3), (-3 - 1 8), (5 - 4 3), (0 - 3 2), (-2 5 4), (1–8 3), (3 4 7), (-8 - 6 2), (5 - 10 4), (1 6 4), (7 - 3 2), (2 - 8 1), (6 0 2), (-1 - 9 4), (-5 4 8), (-7 6 12), (-8 3 0), (-3 2 8) and (-8 0 2) reflections were omitted owing to very bad agreement.

### Crystal data

**Table:**

C_54_H_64_O_12_S_4_	*Z* = 1
*M**_r_* = 1033.29	*F*(000) = 548
Triclinic, *P*1	*D*_x_ = 1.272 Mg m^−^^3^
Hall symbol: -P 1	Mo *K*α radiation, λ = 0.71073 Å
*a* = 10.9366 (6) Å	Cell parameters from 4552 reflections
*b* = 11.7386 (5) Å	θ = 3.6–32.8°
*c* = 12.6262 (8) Å	µ = 0.24 mm^−^^1^
α = 100.018 (4)°	*T* = 293 K
β = 113.884 (6)°	Prism, yellow
γ = 105.884 (4)°	0.49 × 0.32 × 0.14 mm
*V* = 1348.75 (16) Å^3^	

### Data collection

**Table:**

Agilent Xcalibur, Eos, Gemini diffractometer	8908 independent reflections
Radiation source: Enhance (Mo) X-ray Source	6481 reflections with *I* > 2σ(*I*)
Graphite monochromator	*R*_int_ = 0.036
Detector resolution: 16.0416 pixels mm^-1^	θ_max_ = 32.8°, θ_min_ = 3.3°
ω scans	*h* = −14→16
Absorption correction: multi-scan (*CrysAlis PRO*; Agilent, 2014)	*k* = −16→17
*T*_min_ = 0.715, *T*_max_ = 0.967	*l* = −18→17
16365 measured reflections	

### Refinement

**Table:**

Refinement on *F*^2^	0 restraints
Least-squares matrix: full	Hydrogen site location: inferred from neighbouring sites
*R*[*F*^2^ > 2σ(*F*^2^)] = 0.064	H-atom parameters constrained
*wR*(*F*^2^) = 0.169	*w* = 1/[σ^2^(*F*_o_^2^) + (0.0595*P*)^2^ + 1.2423*P*] where *P* = (*F*_o_^2^ + 2*F*_c_^2^)/3
*S* = 1.07	(Δ/σ)_max_ < 0.001
8908 reflections	Δρ_max_ = 0.58 e Å^−^^3^
324 parameters	Δρ_min_ = −0.38 e Å^−^^3^

### Special details

**Table:**

Geometry. Bond distances, angles *etc*. have been calculated using the rounded fractional coordinates. All su's are estimated from the variances of the (full) variance-covariance matrix. The cell e.s.d.'s are taken into account in the estimation of distances, angles and torsion angles
Refinement. Refinement on *F*^2^ for ALL reflections except those flagged by the user for potential systematic errors. Weighted *R*-factors *wR* and all goodnesses of fit *S* are based on *F*^2^, conventional *R*-factors *R* are based on *F*, with *F* set to zero for negative *F*^2^. The observed criterion of *F*^2^ > σ(*F*^2^) is used only for calculating -*R*-factor-obs *etc*. and is not relevant to the choice of reflections for refinement. *R*-factors based on *F*^2^ are statistically about twice as large as those based on *F*, and *R*-factors based on ALL data will be even larger.

### Fractional atomic coordinates and isotropic or equivalent isotropic displacement parameters (Å^2^)

**Table:**

	*x*	*y*	*z*	*U*_iso_*/*U*_eq_	
S1	0.38215 (6)	0.57806 (5)	0.18169 (5)	0.0238 (2)	
S2	0.76046 (6)	0.40179 (5)	0.44376 (5)	0.0202 (1)	
O1	0.49791 (19)	0.46545 (16)	0.38746 (17)	0.0296 (5)	
O2	0.42622 (17)	0.67374 (14)	0.44663 (14)	0.0215 (4)	
O3	0.7778 (2)	0.76578 (19)	0.56813 (17)	0.0372 (6)	
O4	0.6528 (2)	0.86471 (17)	0.62364 (16)	0.0335 (5)	
O5	0.3815 (2)	0.84596 (18)	0.33706 (19)	0.0389 (6)	
O6	0.6192 (2)	0.93356 (16)	0.39687 (19)	0.0357 (6)	
C1	0.6629 (3)	0.7156 (2)	0.2902 (2)	0.0234 (6)	
C2	0.5554 (2)	0.64613 (19)	0.32531 (19)	0.0207 (6)	
C3	0.5857 (2)	0.53717 (19)	0.37028 (19)	0.0207 (6)	
C4	0.7215 (2)	0.52250 (18)	0.38669 (18)	0.0181 (5)	
C5	0.8098 (2)	0.59444 (19)	0.35403 (19)	0.0212 (6)	
C6	0.7789 (2)	0.69103 (19)	0.30120 (19)	0.0208 (6)	
C7	0.8882 (3)	0.7654 (2)	0.2675 (3)	0.0311 (7)	
C8	0.9031 (5)	0.6736 (3)	0.1744 (4)	0.0612 (16)	
C9	0.8420 (4)	0.8595 (3)	0.2101 (4)	0.0574 (14)	
C10	1.0347 (4)	0.8317 (3)	0.3815 (4)	0.0619 (13)	
C11	0.9349 (2)	0.56128 (19)	0.68011 (19)	0.0211 (6)	
C12	0.8017 (2)	0.46496 (19)	0.59880 (18)	0.0188 (5)	
C13	0.2968 (2)	0.58308 (18)	0.35982 (18)	0.0183 (5)	
C14	0.2600 (2)	0.53000 (19)	0.23855 (19)	0.0198 (5)	
C15	0.8742 (2)	0.5656 (2)	0.84102 (19)	0.0227 (6)	
C16	0.9745 (2)	0.61370 (19)	0.8030 (2)	0.0215 (6)	
C17	1.1205 (3)	0.7230 (2)	0.8879 (2)	0.0267 (6)	
C18	1.1203 (3)	0.8358 (3)	0.8437 (3)	0.0508 (10)	
C19	1.2448 (3)	0.6880 (3)	0.8872 (3)	0.0381 (8)	
C20	1.1473 (3)	0.7592 (3)	1.0203 (3)	0.0459 (9)	
C21	0.5354 (2)	0.73515 (19)	0.41950 (19)	0.0206 (6)	
C22	0.6726 (2)	0.7896 (2)	0.5459 (2)	0.0230 (6)	
C23	0.7668 (3)	0.9188 (3)	0.7497 (2)	0.0388 (8)	
C24	0.7386 (4)	0.8350 (4)	0.8208 (3)	0.0584 (11)	
C25	0.4997 (3)	0.8438 (2)	0.3777 (2)	0.0254 (6)	
C26	0.6045 (4)	1.0460 (2)	0.3669 (3)	0.0421 (9)	
C27	0.5772 (5)	1.0344 (4)	0.2411 (4)	0.0649 (16)	
H1	0.64760	0.77950	0.25890	0.0280*	
H5	0.89410	0.58210	0.36560	0.0250*	
H8A	0.97220	0.71940	0.15320	0.0920*	
H8B	0.81100	0.62980	0.10200	0.0920*	
H8C	0.93540	0.61450	0.20990	0.0920*	
H9A	0.83400	0.91880	0.26720	0.0860*	
H9B	0.74990	0.81680	0.13740	0.0860*	
H9C	0.91270	0.90270	0.18920	0.0860*	
H10A	1.10450	0.87590	0.36030	0.0930*	
H10B	1.06450	0.77120	0.41620	0.0930*	
H10C	1.02770	0.89000	0.44030	0.0930*	
H11	0.99940	0.59170	0.65170	0.0250*	
H15	0.89760	0.59850	0.92240	0.0270*	
H18A	1.04430	0.85990	0.84670	0.0760*	
H18B	1.10450	0.81440	0.76110	0.0760*	
H18C	1.21180	0.90420	0.89550	0.0760*	
H19A	1.23340	0.66930	0.80590	0.0570*	
H19B	1.24400	0.61590	0.91340	0.0570*	
H19C	1.33510	0.75680	0.94200	0.0570*	
H20A	1.13890	0.68680	1.04660	0.0690*	
H20B	1.07720	0.79130	1.02490	0.0690*	
H20C	1.24280	0.82220	1.07240	0.0690*	
H23A	0.77040	1.00060	0.78550	0.0460*	
H23B	0.85940	0.92930	0.75290	0.0460*	
H24A	0.81310	0.87190	0.90470	0.0870*	
H24B	0.73800	0.75500	0.78670	0.0870*	
H24C	0.64640	0.82430	0.81680	0.0870*	
H26A	0.52530	1.05910	0.37660	0.0510*	
H26B	0.69250	1.11830	0.42330	0.0510*	
H27A	0.56900	1.10930	0.22420	0.0980*	
H27B	0.48880	0.96410	0.18510	0.0980*	
H27C	0.65590	1.02200	0.23160	0.0980*	

### Atomic displacement parameters (Å^2^)

**Table:**

	*U*^11^	*U*^22^	*U*^33^	*U*^12^	*U*^13^	*U*^23^
S1	0.0226 (3)	0.0322 (3)	0.0206 (2)	0.0101 (2)	0.0132 (2)	0.0111 (2)
S2	0.0258 (3)	0.0211 (2)	0.0220 (2)	0.0135 (2)	0.0152 (2)	0.0090 (2)
O1	0.0275 (9)	0.0328 (8)	0.0439 (10)	0.0162 (7)	0.0236 (8)	0.0235 (7)
O2	0.0186 (7)	0.0234 (7)	0.0220 (7)	0.0053 (6)	0.0113 (6)	0.0066 (6)
O3	0.0275 (9)	0.0493 (11)	0.0312 (9)	0.0218 (8)	0.0096 (8)	0.0042 (8)
O4	0.0275 (9)	0.0360 (9)	0.0298 (9)	0.0157 (7)	0.0094 (7)	−0.0006 (7)
O5	0.0309 (10)	0.0413 (10)	0.0516 (12)	0.0237 (8)	0.0174 (9)	0.0208 (9)
O6	0.0345 (10)	0.0260 (8)	0.0558 (12)	0.0153 (7)	0.0238 (9)	0.0225 (8)
C1	0.0257 (11)	0.0233 (10)	0.0301 (11)	0.0105 (8)	0.0183 (9)	0.0143 (8)
C2	0.0197 (10)	0.0225 (9)	0.0244 (10)	0.0099 (8)	0.0123 (8)	0.0106 (8)
C3	0.0223 (10)	0.0222 (9)	0.0248 (10)	0.0113 (8)	0.0146 (8)	0.0113 (8)
C4	0.0204 (9)	0.0182 (8)	0.0208 (9)	0.0092 (7)	0.0130 (8)	0.0072 (7)
C5	0.0213 (10)	0.0233 (9)	0.0254 (10)	0.0104 (8)	0.0151 (8)	0.0092 (8)
C6	0.0219 (10)	0.0208 (9)	0.0235 (10)	0.0067 (7)	0.0146 (8)	0.0088 (7)
C7	0.0292 (12)	0.0331 (12)	0.0461 (14)	0.0133 (10)	0.0268 (11)	0.0227 (11)
C8	0.087 (3)	0.065 (2)	0.083 (3)	0.038 (2)	0.074 (2)	0.0424 (19)
C9	0.0498 (19)	0.066 (2)	0.099 (3)	0.0315 (16)	0.053 (2)	0.064 (2)
C10	0.0328 (17)	0.056 (2)	0.080 (3)	−0.0015 (14)	0.0203 (17)	0.0304 (18)
C11	0.0205 (10)	0.0255 (10)	0.0250 (10)	0.0122 (8)	0.0141 (8)	0.0119 (8)
C12	0.0199 (9)	0.0231 (9)	0.0188 (9)	0.0120 (7)	0.0103 (8)	0.0104 (7)
C13	0.0182 (9)	0.0205 (9)	0.0206 (9)	0.0104 (7)	0.0103 (8)	0.0092 (7)
C14	0.0189 (10)	0.0249 (9)	0.0210 (9)	0.0106 (8)	0.0117 (8)	0.0106 (7)
C15	0.0227 (10)	0.0277 (10)	0.0199 (9)	0.0118 (8)	0.0105 (8)	0.0084 (8)
C16	0.0191 (10)	0.0221 (9)	0.0241 (10)	0.0091 (8)	0.0101 (8)	0.0076 (8)
C17	0.0194 (10)	0.0289 (11)	0.0273 (11)	0.0068 (8)	0.0102 (9)	0.0051 (8)
C18	0.0352 (16)	0.0312 (13)	0.069 (2)	0.0051 (11)	0.0135 (15)	0.0188 (14)
C19	0.0202 (12)	0.0452 (15)	0.0366 (14)	0.0085 (10)	0.0095 (10)	0.0018 (11)
C20	0.0297 (14)	0.0549 (18)	0.0319 (14)	0.0008 (12)	0.0132 (12)	−0.0052 (12)
C21	0.0189 (10)	0.0215 (9)	0.0245 (10)	0.0092 (7)	0.0111 (8)	0.0100 (8)
C22	0.0228 (10)	0.0218 (9)	0.0261 (10)	0.0098 (8)	0.0117 (9)	0.0094 (8)
C23	0.0336 (14)	0.0396 (14)	0.0291 (12)	0.0133 (11)	0.0083 (11)	−0.0026 (10)
C24	0.061 (2)	0.072 (2)	0.0471 (19)	0.0313 (19)	0.0247 (17)	0.0234 (17)
C25	0.0264 (11)	0.0260 (10)	0.0295 (11)	0.0143 (9)	0.0149 (9)	0.0109 (8)
C26	0.0486 (17)	0.0243 (11)	0.0596 (18)	0.0177 (11)	0.0259 (15)	0.0214 (12)
C27	0.090 (3)	0.064 (2)	0.085 (3)	0.046 (2)	0.060 (3)	0.052 (2)

### Geometric parameters (Å, º)

**Table:**

S1—C2	1.844 (2)	C21—C25	1.548 (3)
S1—C14	1.767 (2)	C23—C24	1.495 (5)
S2—C4	1.774 (2)	C26—C27	1.466 (6)
S2—C12	1.784 (2)	C1—H1	0.9300
O1—C3	1.211 (3)	C5—H5	0.9300
O2—C13	1.360 (3)	C8—H8A	0.9600
O2—C21	1.417 (3)	C8—H8B	0.9600
O3—C22	1.193 (3)	C8—H8C	0.9600
O4—C22	1.328 (3)	C9—H9A	0.9600
O4—C23	1.449 (3)	C9—H9B	0.9600
O5—C25	1.192 (4)	C9—H9C	0.9600
O6—C25	1.334 (4)	C10—H10A	0.9600
O6—C26	1.467 (3)	C10—H10B	0.9600
C1—C2	1.506 (4)	C10—H10C	0.9600
C1—C6	1.336 (4)	C11—H11	0.9300
C2—C3	1.549 (3)	C15—H15	0.9300
C2—C21	1.568 (3)	C18—H18A	0.9600
C3—C4	1.477 (3)	C18—H18B	0.9600
C4—C5	1.347 (3)	C18—H18C	0.9600
C5—C6	1.461 (3)	C19—H19A	0.9600
C6—C7	1.530 (4)	C19—H19B	0.9600
C7—C8	1.543 (6)	C19—H19C	0.9600
C7—C9	1.513 (5)	C20—H20A	0.9600
C7—C10	1.518 (6)	C20—H20B	0.9600
C11—C12	1.392 (3)	C20—H20C	0.9600
C11—C16	1.397 (3)	C23—H23A	0.9700
C12—C13^i^	1.406 (3)	C23—H23B	0.9700
C13—C14	1.388 (3)	C24—H24A	0.9600
C14—C15^i^	1.391 (3)	C24—H24B	0.9600
C15—C16	1.391 (3)	C24—H24C	0.9600
C16—C17	1.536 (4)	C26—H26A	0.9700
C17—C18	1.524 (4)	C26—H26B	0.9700
C17—C19	1.527 (5)	C27—H27A	0.9600
C17—C20	1.533 (4)	C27—H27B	0.9600
C21—C22	1.545 (3)	C27—H27C	0.9600
			
C2—S1—C14	100.38 (10)	C4—C5—H5	119.00
C4—S2—C12	100.21 (10)	C6—C5—H5	119.00
C13—O2—C21	122.89 (17)	C7—C8—H8A	109.00
C22—O4—C23	117.2 (2)	C7—C8—H8B	110.00
C25—O6—C26	117.2 (3)	C7—C8—H8C	109.00
C2—C1—C6	124.0 (2)	H8A—C8—H8B	109.00
S1—C2—C1	104.10 (15)	H8A—C8—H8C	109.00
S1—C2—C3	107.77 (15)	H8B—C8—H8C	109.00
S1—C2—C21	106.81 (16)	C7—C9—H9A	109.00
C1—C2—C3	114.2 (2)	C7—C9—H9B	110.00
C1—C2—C21	112.76 (19)	C7—C9—H9C	109.00
C3—C2—C21	110.56 (17)	H9A—C9—H9B	109.00
O1—C3—C2	120.2 (2)	H9A—C9—H9C	109.00
O1—C3—C4	122.2 (2)	H9B—C9—H9C	109.00
C2—C3—C4	117.6 (2)	C7—C10—H10A	110.00
S2—C4—C3	117.90 (16)	C7—C10—H10B	109.00
S2—C4—C5	120.84 (19)	C7—C10—H10C	109.00
C3—C4—C5	121.2 (2)	H10A—C10—H10B	110.00
C4—C5—C6	122.9 (2)	H10A—C10—H10C	109.00
C1—C6—C5	119.7 (2)	H10B—C10—H10C	109.00
C1—C6—C7	122.8 (2)	C12—C11—H11	119.00
C5—C6—C7	117.4 (2)	C16—C11—H11	119.00
C6—C7—C8	108.9 (2)	C16—C15—H15	119.00
C6—C7—C9	112.4 (3)	C14^i^—C15—H15	119.00
C6—C7—C10	109.2 (3)	C17—C18—H18A	109.00
C8—C7—C9	108.0 (3)	C17—C18—H18B	110.00
C8—C7—C10	108.3 (3)	C17—C18—H18C	109.00
C9—C7—C10	110.0 (3)	H18A—C18—H18B	109.00
C12—C11—C16	122.0 (2)	H18A—C18—H18C	109.00
S2—C12—C11	119.17 (18)	H18B—C18—H18C	110.00
S2—C12—C13^i^	120.95 (16)	C17—C19—H19A	109.00
C11—C12—C13^i^	119.87 (19)	C17—C19—H19B	109.00
O2—C13—C14	125.4 (2)	C17—C19—H19C	109.00
O2—C13—C12^i^	115.73 (18)	H19A—C19—H19B	110.00
C12^i^—C13—C14	118.7 (2)	H19A—C19—H19C	110.00
S1—C14—C13	121.56 (18)	H19B—C19—H19C	109.00
S1—C14—C15^i^	118.17 (16)	C17—C20—H20A	110.00
C13—C14—C15^i^	120.3 (2)	C17—C20—H20B	109.00
C14^i^—C15—C16	122.2 (2)	C17—C20—H20C	109.00
C11—C16—C15	116.9 (2)	H20A—C20—H20B	110.00
C11—C16—C17	120.0 (2)	H20A—C20—H20C	109.00
C15—C16—C17	123.1 (2)	H20B—C20—H20C	109.00
C16—C17—C18	108.9 (2)	O4—C23—H23A	110.00
C16—C17—C19	110.7 (2)	O4—C23—H23B	110.00
C16—C17—C20	111.3 (2)	C24—C23—H23A	110.00
C18—C17—C19	109.2 (3)	C24—C23—H23B	110.00
C18—C17—C20	108.8 (2)	H23A—C23—H23B	108.00
C19—C17—C20	107.8 (2)	C23—C24—H24A	110.00
O2—C21—C2	114.06 (18)	C23—C24—H24B	110.00
O2—C21—C22	102.53 (17)	C23—C24—H24C	109.00
O2—C21—C25	106.9 (2)	H24A—C24—H24B	110.00
C2—C21—C22	110.37 (19)	H24A—C24—H24C	109.00
C2—C21—C25	113.09 (18)	H24B—C24—H24C	109.00
C22—C21—C25	109.30 (18)	O6—C26—H26A	109.00
O3—C22—O4	126.5 (2)	O6—C26—H26B	109.00
O3—C22—C21	124.4 (2)	C27—C26—H26A	109.00
O4—C22—C21	109.1 (2)	C27—C26—H26B	109.00
O4—C23—C24	109.6 (3)	H26A—C26—H26B	108.00
O5—C25—O6	125.9 (2)	C26—C27—H27A	109.00
O5—C25—C21	123.6 (2)	C26—C27—H27B	109.00
O6—C25—C21	110.5 (3)	C26—C27—H27C	109.00
O6—C26—C27	111.2 (3)	H27A—C27—H27B	109.00
C2—C1—H1	118.00	H27A—C27—H27C	109.00
C6—C1—H1	118.00	H27B—C27—H27C	109.00
			
C14—S1—C2—C21	−48.10 (17)	C3—C4—C5—C6	0.2 (3)
C2—S1—C14—C13	22.1 (2)	S2—C4—C5—C6	177.04 (16)
C2—S1—C14—C15^i^	−156.25 (19)	C4—C5—C6—C7	−179.6 (2)
C14—S1—C2—C1	−167.61 (17)	C4—C5—C6—C1	3.9 (3)
C14—S1—C2—C3	70.72 (17)	C5—C6—C7—C10	−59.5 (3)
C12—S2—C4—C5	111.75 (19)	C1—C6—C7—C8	−125.1 (3)
C4—S2—C12—C13^i^	105.5 (2)	C1—C6—C7—C10	116.9 (3)
C12—S2—C4—C3	−71.32 (18)	C5—C6—C7—C9	178.2 (3)
C4—S2—C12—C11	−76.0 (2)	C5—C6—C7—C8	58.5 (3)
C13—O2—C21—C25	84.7 (2)	C1—C6—C7—C9	−5.4 (4)
C13—O2—C21—C2	−41.1 (3)	C16—C11—C12—S2	−179.19 (18)
C13—O2—C21—C22	−160.4 (2)	C16—C11—C12—C13^i^	−0.6 (4)
C21—O2—C13—C14	7.0 (3)	C12—C11—C16—C15	−0.3 (3)
C21—O2—C13—C12^i^	−177.6 (2)	C12—C11—C16—C17	−177.8 (2)
C23—O4—C22—O3	−3.3 (4)	S2—C12—C13^i^—O2^i^	−3.9 (3)
C22—O4—C23—C24	−90.8 (3)	C11—C12—C13^i^—C14^i^	2.0 (3)
C23—O4—C22—C21	176.2 (2)	S2—C12—C13^i^—C14^i^	−179.51 (18)
C25—O6—C26—C27	−92.1 (4)	C11—C12—C13^i^—O2^i^	177.6 (2)
C26—O6—C25—C21	−177.4 (2)	O2—C13—C14—S1	−0.7 (3)
C26—O6—C25—O5	0.9 (4)	C12^i^—C13—C14—C15^i^	2.4 (3)
C6—C1—C2—C21	124.4 (2)	C12^i^—C13—C14—S1	−175.92 (17)
C6—C1—C2—C3	−2.9 (3)	O2—C13—C14—C15^i^	177.6 (2)
C2—C1—C6—C7	−178.5 (2)	S1—C14—C15^i^—C16^i^	176.83 (19)
C2—C1—C6—C5	−2.2 (3)	C13—C14—C15^i^—C16^i^	−1.6 (4)
C6—C1—C2—S1	−120.2 (2)	C14^i^—C15—C16—C11	−0.2 (4)
C1—C2—C21—O2	175.07 (19)	C14^i^—C15—C16—C17	177.3 (2)
C1—C2—C3—O1	−171.4 (2)	C15—C16—C17—C20	7.1 (4)
S1—C2—C3—C4	121.69 (18)	C15—C16—C17—C18	−112.9 (3)
C1—C2—C3—C4	6.6 (3)	C15—C16—C17—C19	127.0 (3)
S1—C2—C21—C22	176.10 (15)	C11—C16—C17—C19	−55.6 (3)
S1—C2—C21—C25	−61.1 (2)	C11—C16—C17—C20	−175.5 (2)
S1—C2—C3—O1	−56.3 (2)	C11—C16—C17—C18	64.5 (3)
C3—C2—C21—C25	−178.1 (2)	C2—C21—C22—O4	179.86 (19)
C21—C2—C3—C4	−121.9 (2)	C25—C21—C22—O3	−125.7 (3)
C1—C2—C21—C22	−70.1 (2)	C2—C21—C25—O5	103.3 (3)
S1—C2—C21—O2	61.3 (2)	C2—C21—C25—O6	−78.3 (2)
C3—C2—C21—C22	59.1 (2)	C22—C21—C25—O5	−133.3 (2)
C1—C2—C21—C25	52.7 (3)	C22—C21—C25—O6	45.1 (3)
C3—C2—C21—O2	−55.7 (2)	C25—C21—C22—O4	54.9 (3)
C21—C2—C3—O1	60.2 (3)	O2—C21—C25—O5	−23.0 (3)
C2—C3—C4—C5	−5.5 (3)	O2—C21—C25—O6	155.34 (19)
C2—C3—C4—S2	177.62 (15)	O2—C21—C22—O3	121.2 (3)
O1—C3—C4—C5	172.4 (2)	O2—C21—C22—O4	−58.3 (2)
O1—C3—C4—S2	−4.5 (3)	C2—C21—C22—O3	−0.7 (3)

Symmetry code: (i) −*x*+1, −*y*+1, −*z*+1.

### Hydrogen-bond geometry (Å, º)

**Table:**

*D*—H···*A*	*D*—H	H···*A*	*D*···*A*	*D*—H···*A*
C26—H26*A*···O4^ii^	0.97	2.35	3.298 (5)	164

Symmetry code: (ii) −*x*+1, −*y*+2, −*z*+1.
