# Supplementary figures and images for: Crystal structure of tetra­ethyl 27,30-dioxo-7,12,20,25-tetra-tert-but­yl-3,16-dioxa-9,22,28,31-tetra­thia­hepta­cyclo­[21.3.1.11,5.14,8.110,14.114,18.117,21]dotriaconta-4,6,8(29),10,12,17,19,21(32),23,25-deca­ene-2,2,15,15-tetra­carboxyl­ate
Source: Acta Crystallogr E Crystallogr Commun. 2015 Sep 26;71(Pt 10):o778–9. doi: 10.1107/S205698901501748X (PMC4647429; doi:10.1107/S205698901501748X)

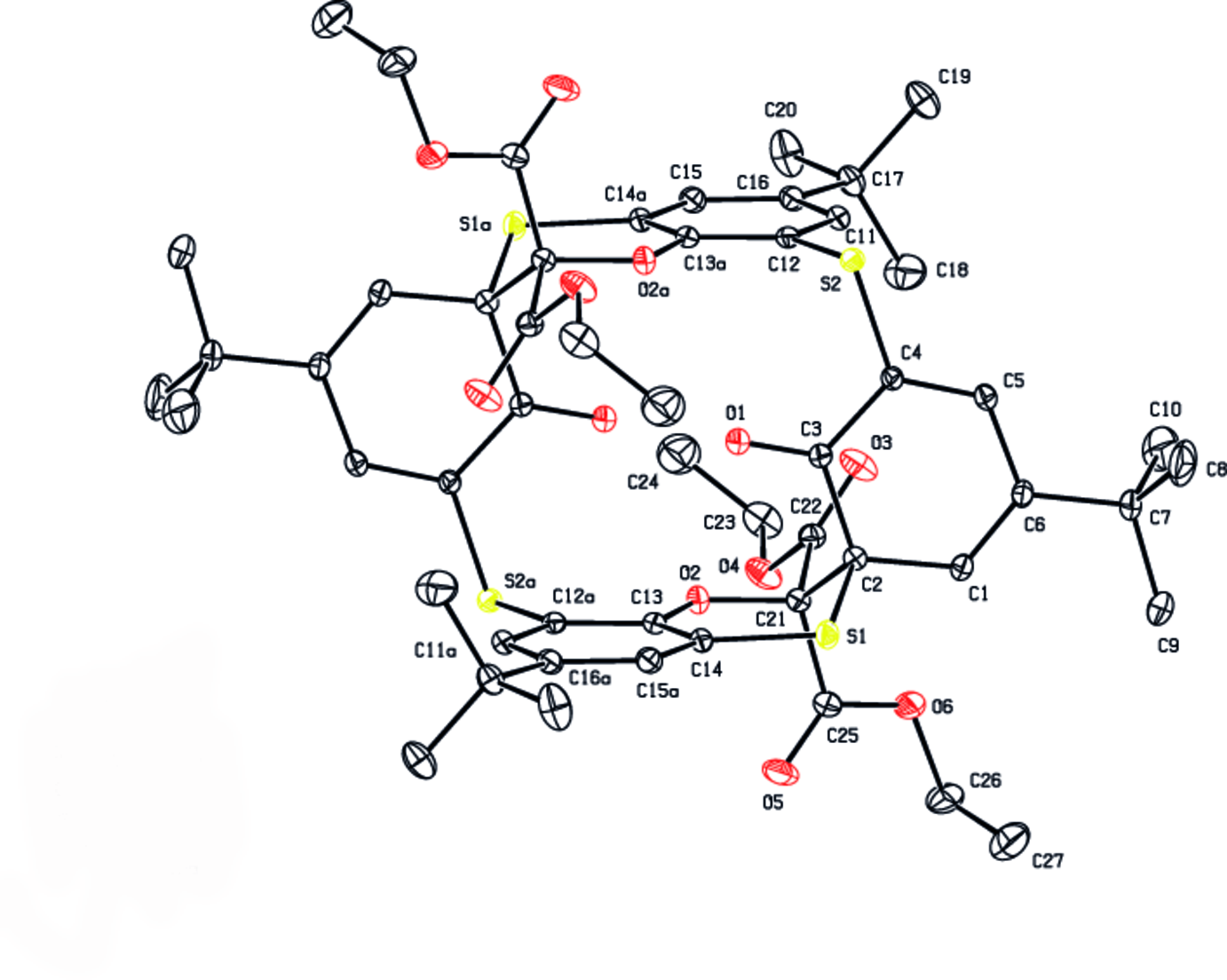

Supplement: Supplementary file 3 [file e-71-0o778-fig1.tif]

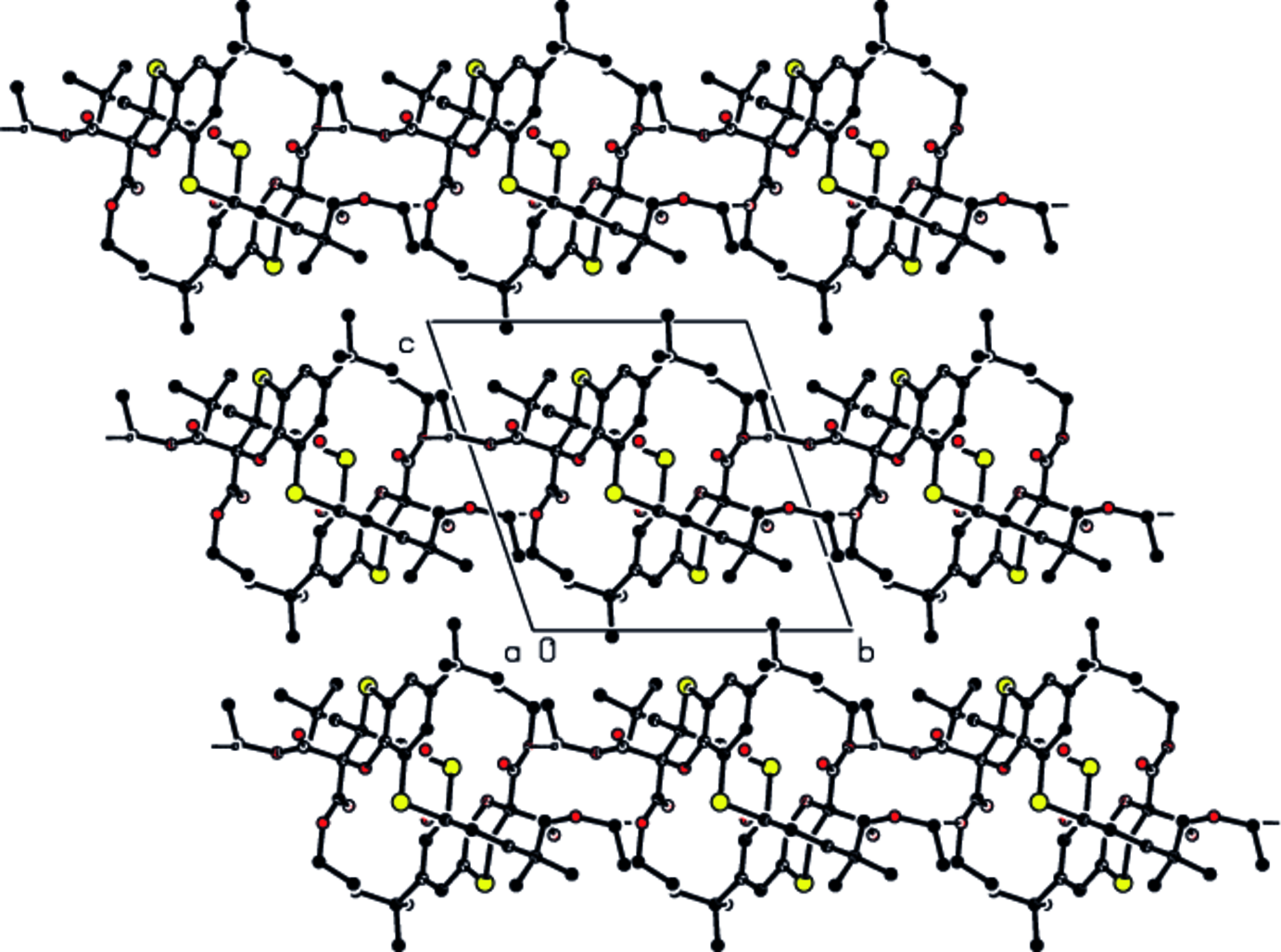

Supplement: Supplementary file 4 [file e-71-0o778-fig2.tif]
